# Supplementary material for: The Drosophila mauritiana synaptonemal complex protein C(3)G repatterns the recombination landscape of Drosophila melanogaster
Source: PLoS Genet. 2025 Sep 23;21(9):e1011882. doi: 10.1371/journal.pgen.1011882 (PMC12478922; doi:10.1371/journal.pgen.1011882)
Supplement: S1 Fig — The codon optimized nucleotide D. mauritiana c(3)G coding sequence (line 1) is shown aligned with publicly available nucleotide sequences for D. mauritiana (line 2) and D. melanogaster c(3)G (line 3). Amino acid sequence coded by each codon is also displayed illustrating that the codon optimized nucleotide sequence retained the amino acid sequence of the publicly available D. mauritiana sequence. (PDF) [file pgen.1011882.s001.pdf]

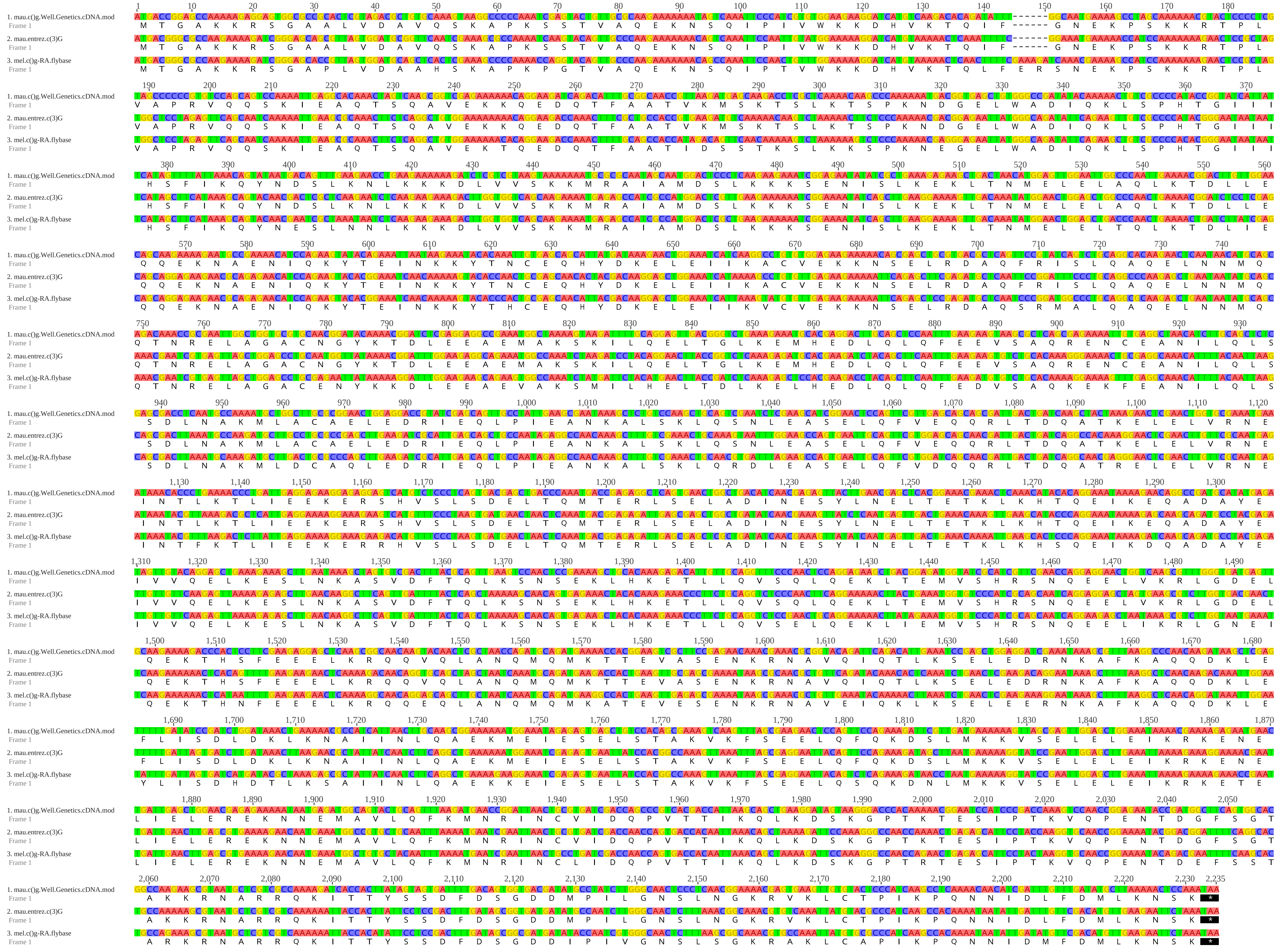
1. mau.c()g.Well.Genetics.cDNA.mod
2. mau.entrez.c(3)G
3. mel.c()g-RA.flybase
Frame 1
