# Supplementary figures and images for: The Drosophila mauritiana synaptonemal complex protein C(3)G repatterns the recombination landscape of Drosophila melanogaster
Source: PLoS Genet. 2025 Sep 23;21(9):e1011882. doi: 10.1371/journal.pgen.1011882 (PMC12478922; doi:10.1371/journal.pgen.1011882)

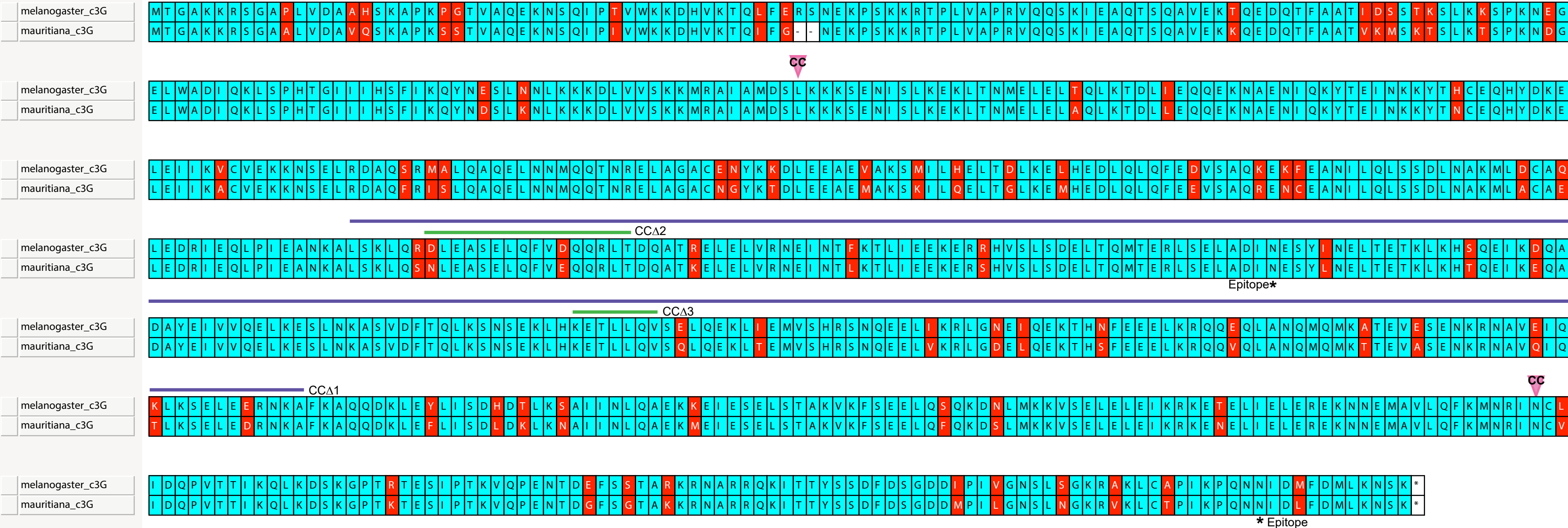

Supplement: S2 Fig — Amino acid differences are displayed in red and gaps in white. The proteins show 89.1% identity with differences dispersed throughout the protein. The C(3)G protein is predicted to have a large coiled-coil domain in the center of the protein. The predicted start and end of the coiled-coil domain is indicated on the D. melanogaster sequence by pink triangles. Green and purple lines just above the D. melanogaster sequence indicate the locations of the three deletion mutations described in Billmyre et al. [43]. Asterisks below the D. mauritiana sequence indicate the start and end of the region expressed to generate the D. mauritiana C(3)G antibody. (PDF) [file pgen.1011882.s002.pdf]

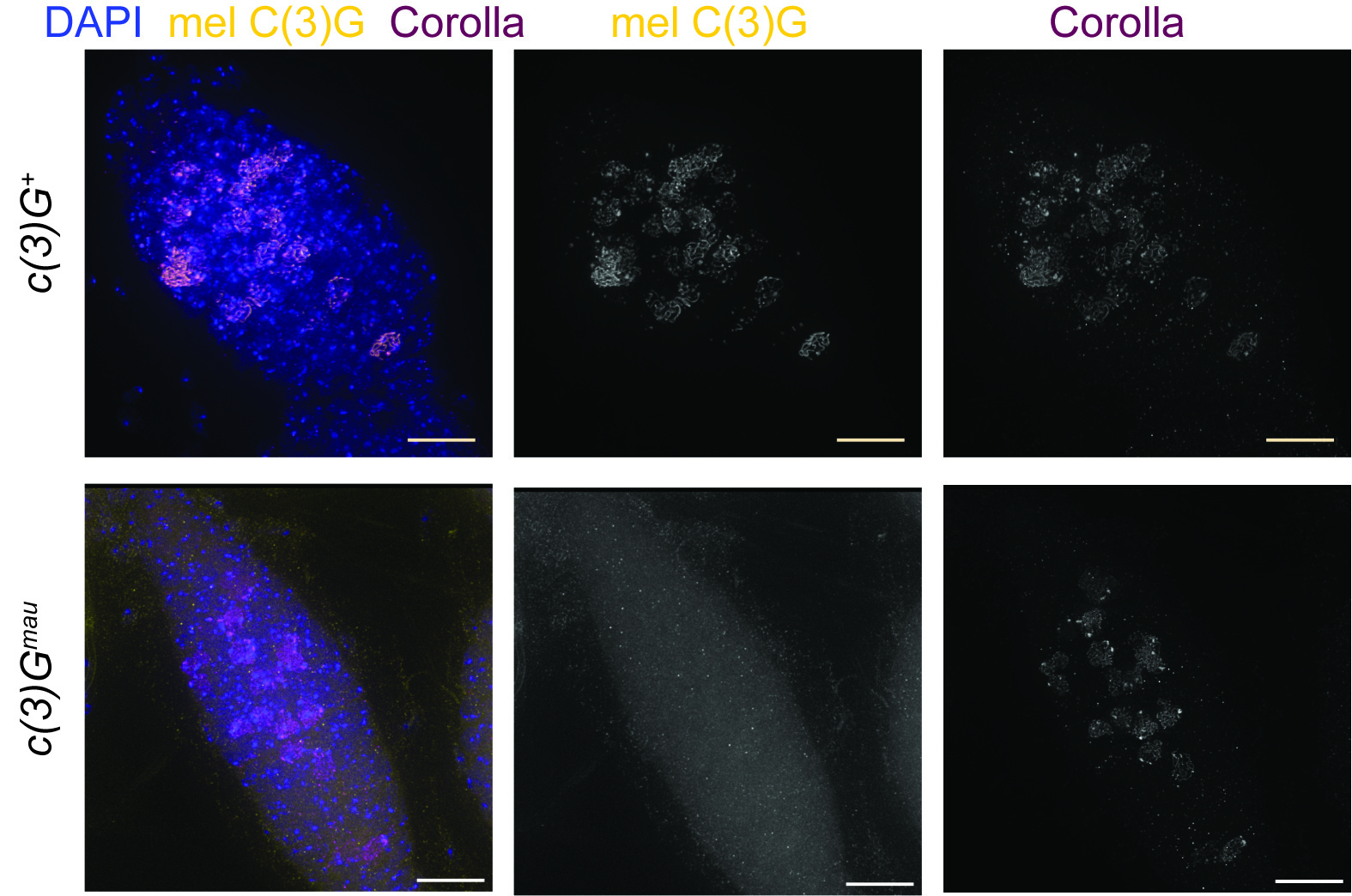

Supplement: S3 Fig — Germaria from c(3)G+ (top) and c(3)Gmau (bottom) stained with antibodies recognizing the C-terminus of the D. melanogaster C(3)G protein (green) and Corolla (magenta).While the antibodies show overlapping localization in c(3)G+, the D. melanogaster C(3)G antibody does not show colocalization with Corolla in c(3)Gmau. Images are projections from z-stacks and scale bar = 10 µm. (TIF) [file pgen.1011882.s003.tif]

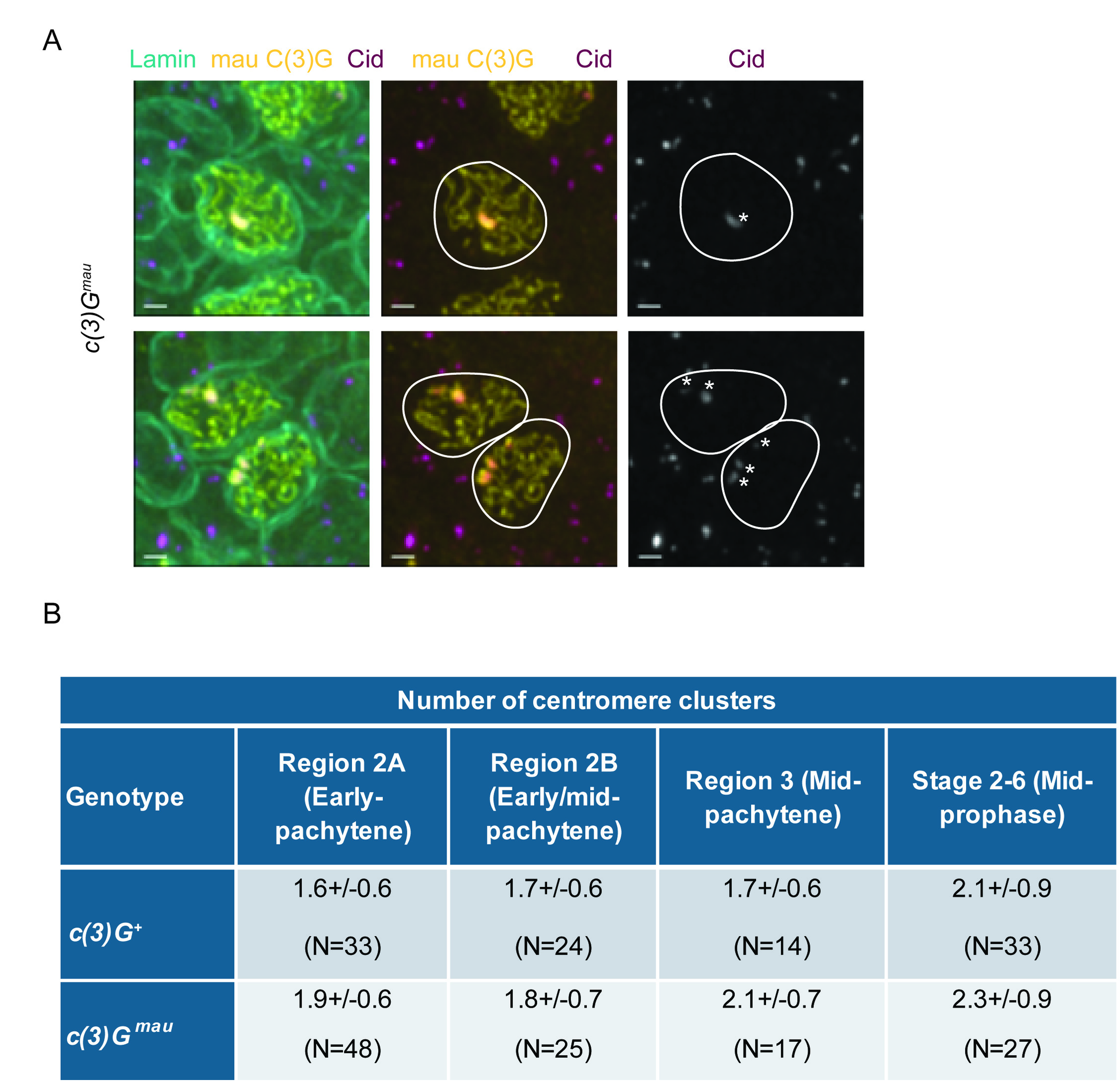

Supplement: S4 Fig — (A) Examples of nuclei from c(3)Gmau ovaries with 1 (top row), 2, or 3 (bottom row) centromere clusters. Centromeres are labeled with an antibody recognizing the centromeric histone CID (magenta), mau C(3)G antibody labels the SC (yellow), and lamin antibody outlines the nuclear envelope (cyan). Asterisks indicate the location of centromere clusters. Scale bar = 1 µm and images are projections from partial z-stacks. (B) The average number of centromere clusters with standard deviation at the indicated developmental stages in c(3)G+ and c(3)Gmau ovaries. Number of nuclei scored in parentheses. Mann-Whitney U test found no statistically significant difference between the genotypes for any of the stages examined (p > 0.05). (TIF) [file pgen.1011882.s004.tif]

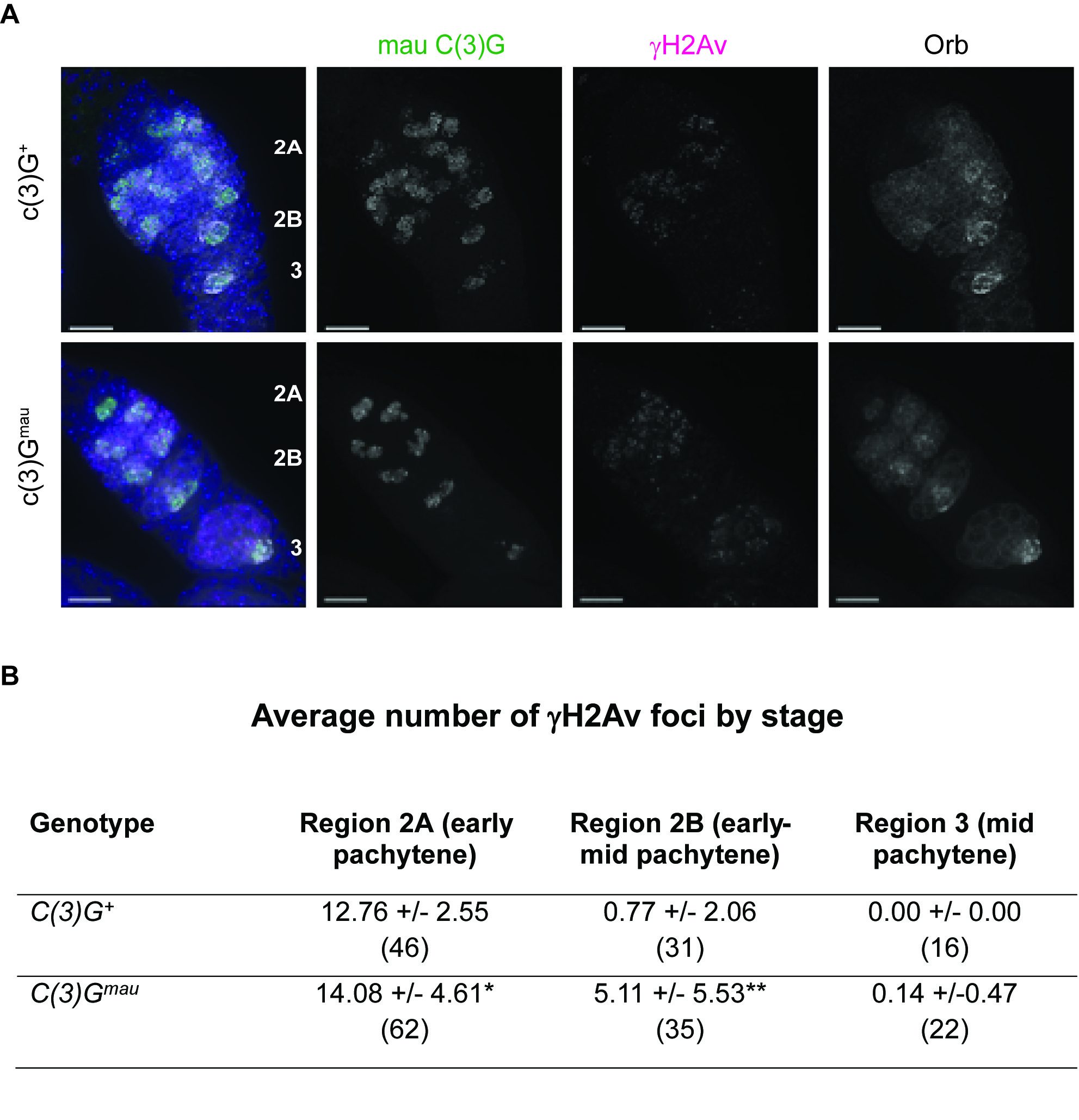

Supplement: S5 Fig — (A) c(3)G+ (top) and c(3)Gmau (bottom) germaria were stained with antibodies to mau C(3)G to identify the SC (green), γH2Av to recognize DSBs (magenta), Orb to stage oocyte development (white), and DAPI (blue). In both genotypes DSBs are induced in region 2A (early pachytene) and γH2Av staining is absent from the Orb selected nucleus in region 3 (mid pachytene). Note in the c(3)Gmau image γH2Av foci are present in the nurse cells that have started endoreduplication in region 3. Scale bar = 10 µm and images are projections from z stacks. (B) Table provides the average number with standard deviation of γH2Av foci for each region of the germarium. The total number of nuclei scored is in parentheses. By Mann-Whitney U test the statistically significant differences from c(3)G+ are indicated as * p < 0.05 and **p < 0.01. Full genotypes are y w;; c(3)G+; spapol and y w;; c(3)Gmau; spapol. (TIF) [file pgen.1011882.s005.tif]
