# Supplementary material for: The Drosophila mauritiana synaptonemal complex protein C(3)G repatterns the recombination landscape of Drosophila melanogaster
Source: PLoS Genet. 2025 Sep 23;21(9):e1011882. doi: 10.1371/journal.pgen.1011882 (PMC12478922; doi:10.1371/journal.pgen.1011882)
Supplement: Attachment — Submitted filename: response to reviewers Hughes et al.docx [file pgen.1011882.s007.docx]

PGENETICS-D-25-00642
The recombination landscape of Drosophila melanogaster can be repatterned by a single gene


Dear editors,

We are grateful for the time and attention the reviewers and editors devoted to our manuscript. We are pleased by the reviewers’ enthusiasm for our work and value their insightful recommendations for improving the manuscript. We have modified the manuscript to comply as fully as possible with the reviewers’ concerns, and a detailed list of those changes is provided below.

A summary of the major changes to the manuscript includes incorporating the first paragraphs of the results into the introduction as suggested by reviewer #1. As suggested by reviewer #2 we have updated the title, streamlined the text, and provided the requested caveat in the discussion. We provided the figure updates indicated by reviewer #3.

We thank you in advance for your time and effort. We hope you will find this revised version of the manuscript suitable for publication.

Sincerely,

Stacie Hughes Ph.D. 


 

**Reviewers' comments: (*responses in italics*)**

Reviewer #1: The manuscript by Hugues et al. explores the consequences of replacing the central element protein C(3G) from D. melanogaster with its ortholog from D. mauritana. The data demonstrate that D. mauritana C(3G) is functional in D. melanogaster and supports all meiotic functions. Interestingly, this results in a significant increase in crossover frequency in pericentromeric intervals, suggesting that C(3)G supports the centromere effect (suppression of CO) and that variations in this protein can generate interspecific differences in crossover distribution.
The manuscript is well organized, and I only have some suggestions to improve clarity.

On line 95, define "centromere" and describe its structure.

*We have provided a brief definition and description of the Drosophila melanogaster centromere, as well as a reference describing the centromeres in detail.*

Line 103: Clarify/correct "increased level of suppression."

*We have changed the sentence to “decreased levels of crossing over in those regions”.*

Lines 123–135: Improve the readability of this sentence.

*The sentence has been broken up into multiple sentences and changed to try to improve readability.*

Results: The first two to three paragraphs should be included in the introduction.

*Parts of the beginning of the results were incorporated throughout the introduction.*

Line 218: Clarify/specify "similar genetic background"

*We have changed the text to indicate that for these studies c(3)G^mau^ was compared to a stock containing the same marked chromosomes but with the original melanogaster c(3)G. As different experiments required the addition of different genetic markers, we strived to only compare the replacement line to a line with the same marked chromosomes to minimize any additional genetic differences between the strains.*

L233-234 is unclear. Do you mean that no signal was detected in C(3)Gmau fly oocytes?

*We have now included a new supplementary figure S3 showing that while the melanogaster C(3)G C-terminal antibody used in prior publications localizes with Corolla in c(3)G^+^ flies it fails to localize to the SC in c(3)G^mau^ ovaries.*

L251. Define "n."

*N is the number of measurements taken. N was defined in the legend of Figure 3, but we have now also added “measurement” at the first use in text.*

L324: "DSB foci" should be "gH2Av foci."

*Thank you for highlighting this. As the γH2Av antibody may not recognize DSBs at later stages of repair we tried to emphasize we were examining γH2Av in the text but appeared to have interchanged the two terms in a couple of places. These have now been corrected.*

L380 and Tables. What are the "rate of crossing over" and "map length"? Is it simply a fraction of recombined chromatids or another calculation?

*It is the number of recombinant chromatids divided by total progeny and multiplied by 100 for easier comparisons. This is now defined in both the table footnotes and text.*

L532-533. "A mutation promotes SC assembly." This should be rephrased.

*The sentence was rephrased.*

Reviewer #2: This paper, by Stacie Hughes, the late Scott Hawley and colleagues addresses the potential role of C(3)G - the fly transverse filament protein - in regulating the crossover landscape. Specifically, they test the hypothesis that divergence in the amino acid sequence of C(3)G between D. melanogaster and D. mauritania is responsible for differences in the crossover landscape between these two closely related species. They do that by precise gene replacement of C(3)G and analysis of the recombination landscape. They indeed identified a relatively dramatic change in the crossover landscape that is consistent with the differences observed between the two Drosophila species. Given our still-limited understanding of crossover regulation by the synaptonemal complex, linking a single protein to a specific effect on crossover distribution would constitute an important advance in the field.

The major concern with the manuscript is the conclusion that swapping C(3)G caused the recombination landscape in melanogaster to become more similar to that landscape of Mauritania. While the effect of the swap is consistent with this possibility, there are other potential possibilities. The one most likely in my opinion is that the observed effects are the result of a somewhat malfunctioning SC. This idea is consistent with some similarity with the effect the Hawley lab observed in strains with small deletions in C(3)G (the effect of these small deletions were distinct from the ones observed here, but are not unrelated). It is also consistent with the prolonged duration of H2Av staining. In other words, the observed phenotypes could be the result of some inter-chromosomal effect (analogous to the effect of pairing & synapsis mutants on the CO landscape in worms).

There are two possible ways to resolve this issue. One will be to perform significant amount of additional work to rule out dysfunction of the SC in the swapped strain. That would not be trivial to do, but as a start, more comprehensive analysis to show that the SC is functioning normally (or as normally as could be assessed) would be needed. It will also involve some chasing down of the extended H2Av signal. In addition, the degree of similarity to the D. mauritania CO landscape should be quantified (and, if the claim in relation to the mei-218 swap is maintained, that difference should also be quantified.) The second possibility is to dramatically tone down the claims of this manuscript to better reflect the findings of this paper.

*This is an interesting point. If we are understanding correctly, what the reviewer is saying is that there are two possible reasons that the recombination landscape becomes “mau-like” when C(3)G is replaced with the D. mauritiana version. One possibility is that it establishes a recombination landscape in D. melanogaster through the same mechanism by which C(3)G divergences drives the difference observed in the mau recombination landscape. In other words, the divergence in C(3)G between mel and mau is identically causal (through the same mechanism) for a different recombination rate in mau and mel with C(3)G replaced.*

*Alternatively, the similarity between the native mau recombination landscape and that established in melanogaster through C(3)G replacement could be driven by mere mimicry, rather than the same mechanism. Put more precisely, imagine the difference in recombination between mau and mel is explained by a divergence in mechanism X. However, by replacing the C(3)G of mel with that of mau, some OTHER event arises (say, event Q) and event Q causes a difference in the recombination landscape via mechanism Z. It just so happens that, through mechanism Z, there is apparent mimicry of mechanism X? In this scenario, event Q may be some defect in SC formation that arises in neither wild-type mel nor wild-type mau. Is this what the reviewer is driving at?*

*This is an interesting point. Of course, with respect to a defect in SC formation, we argue that we have been fairly exhaustive in the evaluation of this manner (segregation, centromere clustering, tripartite SC formation, etc.). That being said, we can’t rule out some cryptic event Q.We don’t think this changes the conclusion stated in the title of the paper (which we have revised by request). But, it is a worthwhile point. We now add this discussion to tone down the conclusion. The text added is as follows:*

*“One thing to consider, however, is whether the mechanism, by which replacement of the native D. melanogaster C(3)G with that of D. mauritiana leads to a change in the recombination landscape (to one more similar to D. mauritiana), is identical to the cause of divergence in the recombination landscape between these two species. In fact, the observed transformation to a D. mauritiana-like recombination landscape may instead occur through an alternative mechanism, and the similarity may be a case of mere coincidental similarity. For example, if there is some cryptic disruption in SC function that occurs in the D. melanogaster replacement line, but in neither of the two species, this could trigger some event that leads to a change in recombination through an alternative mechanism. We do not believe this is likely to be the case, because the SC phenotype was exhaustively investigated and only marginal differences were found. Still, we cannot completely rule out the possibility that the similarity of the recombination landscape to that of wild-type D. mauritiana in the replacement line is through a different mechanism than the one that causes the differences between the two species.”*

*Brand et al provided no cytological data for the Mei-218 experiments. Joyce et al 2012 showed in an image that a mei-218 loss-of-function mutant forms SC. As SC formation is not dependent on DSB formation in Drosophila melanogaster females it would not be expected that D. mauritiana Mei-218 would greatly affect SC formation and likely affects crossover placement through a different mechanism. The SC has been proposed to affect crossover placement and number by affecting the coarsening of pro-crossover factors. Mei-218 has been proposed to work with other pro-crossover factors, and it seems more likely D. mauritiana Mei-218 changes the localization of itself or other repair proteins to repair sites. Data from Durand et al 2022 of Arabidopsis indicate crossover patterning is a combination of both pro-crossover machinery and the SC as Hei-10 overexpression and loss of the SC both increased crossing over and the effects were additive. Therefore, two mechanisms regulating crossover patterning in Drosophila are possible. We do not feel we should speculate further on the molecular function of Mei-218 in this manuscript.*

*There is not much additional information that can be added about the γH2Av foci in region 2B. While regions 2A and 3 appear to have more consistent values across studies (many foci in region 2A and nearly zero in region 3) the values reported for wild-type females can vary at region 2B. Mehrotra and McKim reported an average of 8.5 foci for early 2B and 3.5 foci for late region 2B while Lake et al. 2013 reported an average of 3.8 foci for all of region 2B. We have now expanded the sentence to highlight that variability. Additionally, mutants with dysfunctional SC, as in Billmyre et al. 2019 and Mehrotra and McKim 2006, do not display higher numbers of H2Av foci than controls at region 2B and loss of SC cause a decrease in foci at region 2A.*

The other concerns are more minor. The title of the manuscript, which does not reflect the novelty of this work, should be edited. As mentioned above, multiple single-gene alterations were shown to affect the recombination landscape. A more descriptive title would be more appropriate.

*We have updated the title to “*The *Drosophila mauritiana* synaptonemal complex protein C(3)G repatterns the recombination landscape of *Drosophila melanogaster.”*

In addition, the text and figures would benefit from significant streamlining. It is more appropriate as a report- or letter-style format. (I realize PLoS Genetics doesn’t formally have such a format, but aiming for the equivalent of that would be ideal.)

*While still addressing the requests of the other reviewers we have tried to streamline the manuscript.*

Reviewer #3: Over the past ~10 years, the role of the synaptonemal complex in crossover regulation has been an emergent theme among crossover regulation papers across organisms. This manuscript by Hughes et al. takes the approach of replacing the transverse filament SC component C(3)G in Drosophila melanogaster with that of Drosophila mauritiana, in which it is known to have decreased crossover number and patterning. Prior work has shown that replacement of D. melanogaster mei-218 with that of D. mauritiana found that crossovers specifically in the telomeres and centromeres were increasing. By performing this elegant approach with C(3)G, Hughes et al. were able to recapitulate the crossover landscape in D. mauritiana for the intervals they tested across several chromosomes. This work not only emphasizes the importance of the SC and the transverse filament in crossover regulation, but also starts to identify the molecular mechanisms behind this regulation of the SC in crossover regulation (e.g. how the width and specific residues may affect the propagation of an interference ‘signal’ within the SC) as well as solidify the fact crossover regulation (and perhaps interference) is controlled by multiple factors. I only have minor comments:
1. The paper both strongly builds and supports the findings and suggestions of the Billmyre and Cahoon et al., PNAS 2019 paper where they made internal in frame deletions of the coiled-coil domain of D. melanogaster C(3)G and found specific effects on recombination for each deletion. The conclusions and interpretations of the present study would be strengthened by indicating where the amino acid differences in C(3)G between D. melanogaster and D. mauritiana map to the internal deletions made in the Billmyre 2019 paper. Figure S2 or an additional figure could be generated to indicate where the structure domains of C(3)G are in the two a.a. sequences and where the Billmyre deletions map at least in the melanogaster sequence.

*We have now indicated the location of the deletions created in Billmyre and Cahoon et al. 2019 and the coiled-coil domain in D. melanogaster in S2 Fig.*

2. Whether decreased (or a loss) of crossover interference is required for fertility is an ongoing debate (currently thought to not necessarily be required for fertility), therefore reporting the overall effect on fertility (e.g. brood size and dead egg/embryo) of C(3)G of D. mauritiana in the D. melanogaster where crossover interference is now decreased may increase in the ability of the paper to be cited in the context of future studies that resolve this question of whether the highly conserved process of crossover interference is required for fertility.

*As the level of crossing over in c(3)G^mau^ occurs naturally in the D. mauritiana species we did not initially consider that we would need to discuss this potential issue. Thank you for bringing it to our attention. While past studies in flies, humans, and Arabidopsis have correlated either the increased total recombination or increased centromere proximal crossovers to mild defects in fertility or MII chromosome segregation, the c(3)G^mau^ stocks are healthy when homozygous for the replacement, and the females were quite fertile in the genetic assays. We have now included the fertility of the females from the X recombination data as an example of the fertility of the replacement flies and mention previous experimental data on the subject.*

3. Figure S3 - The study finds that the SC is destabilized in specific stages of the meiosis (e.g. regions of the germarium). Specifically, they find that region 3 (mid-pachytene) may be affected. I think this is an important finding about the regulation/stability of the SC and how specific residues of the transverse filament (or the width of the SC) may impact this. I think Figure S3 should be added to the main figures of the paper so that this finding isn’t missed by readers. For Figure S3, the addition of D. melanogaster example images of the different stages/regions as well as state to indicate the significances of the difference would strength the figure for addition to the main figures.

*We have now included images of full-length SC from c(3)G^+^ and c(3)G^mau^ at region 2A and 2B and a full-length SC image of c(3)G^+^ at region 3 to go with the images of the three observed phenotypes for c(3)G^mau^ at region 3. This figure is now Figure 4.*

4. The study finds that the SC width with the C(3)G of D. mauritiana is thinner than that of D. melanogaster, which is commiserate with a decrease in crossover interference especially in centromere proximal regions. This is a very minor point (especially given differences in genome size, centromere-type, heterochromatin), but how does this specific SC width and interference strength compare with other organisms that have a similar SC width?

*That is an interesting question but not one that is easy to answer. There are only a relatively small number of organisms that have both published recombination data and SC width data available. Another issue is that SC width data is not always acquired in the same fashion. Here we are measuring fluorescence of an antibody made against the C-terminal half of the transverse filament protein while other measurements have been made using EM by either measuring from the inside or outside of the electron dense LE. Additionally, there is a correlation between SC length and level of recombination (longer SCs tend to have more crossovers than shorter SCs). As organisms have different sized chromosomes that are packaged within the SC differently (sometime even packaged differently between the two sexes of a species) to really examine this question corrections to the data would be needed to directly compare diverse species. Due to these complexities, we feel we cannot adequately address this question in the discussion, and it would make for a better topic for a review that can explore all these differences.*
